# Research data explored: an extended analysis of citations and altmetrics

**DOI:** 10.1007/s11192-016-1887-4

**Published:** 2016-02-15

**Authors:** Isabella Peters, Peter Kraker, Elisabeth Lex, Christian Gumpenberger, Juan Gorraiz

**Affiliations:** ZBW Leibniz Information Centre for Economics, Düsternbrooker Weg 120, 24105 Kiel, Germany; Kiel University, Christian-Albrechts-Platz 4, 24118 Kiel, Germany; Know-Center, Inffeldgasse 13, 8010 Graz, Austria; Knowledge Technologies Institute, Graz University of Technology, Inffeldgasse 13, 8010 Graz, Austria; Vienna University Library, Department of Bibliometrics & Publication Strategies, University of Vienna, Boltzmanngasse 5, 1090 Vienna, Austria

**Keywords:** Altmetrics, Citation analysis, Co-citation analysis, Citedness, Research data, Data Citation Index

## Abstract

In this study, we explore the citedness of research data, its distribution over time and its relation to the availability of a digital object identifier (DOI) in the Thomson Reuters database Data Citation Index (DCI). We investigate if cited research data “impacts” the (social) web, reflected by altmetrics scores, and if there is any relationship between the number of citations and the sum of altmetrics scores from various social media platforms. Three tools are used to collect altmetrics scores, namely PlumX, ImpactStory, and Altmetric.com, and the corresponding results are compared. We found that out of the three altmetrics tools, PlumX has the best coverage. Our experiments revealed that research data remain mostly uncited (about 85 %), although there has been an increase in citing data sets published since 2008. The percentage of the number of cited research data with a DOI in DCI has decreased in the last years. Only nine repositories are responsible for research data with DOIs and two or more citations. The number of cited research data with altmetrics “foot-prints” is even lower (4–9 %) but shows a higher coverage of research data from the last decade. In our study, we also found no correlation between the number of citations and the total number of altmetrics scores. Yet, certain data types (i.e. survey, aggregate data, and sequence data) are more often cited and also receive higher altmetrics scores. Additionally, we performed citation and altmetric analyses of all research data published between 2011 and 2013 in four different disciplines covered by the DCI. In general, these results correspond very well with the ones obtained for research data cited at least twice and also show low numbers in citations and in altmetrics. Finally, we observed that there are disciplinary differences in the availability and extent of altmetrics scores.

## Introduction

Recently, data citations have gained momentum (Piwowar and Chapman 2010; Borgman 2012; Torres-Salinas et al. 2013b). This is reflected, among others, in the development of data-level metrics (DLM), an initiative driven by PLOS, UC3 and DataONE,^1^ to track and measure activity on research data, and the recent announcement of CERN to provide digital object identifier (DOIs) for each dataset they share through their novel Open Data portal.^2^ In the latter case, the aim is “to make [data sets] citable objects in the scientific discourse”. Data citations are citations included in the reference list of a published article that formally cite either the data that led to a research result or a data paper.^3^ Thereby, data citations indicate the influence and reuse of data in scientific publications.

First studies on data citations showed that certain well-curated data sets receive far more citations or mentions in other articles than many traditional articles (Belter 2014; Parsons et al. 2010; Piwowar et al. 2007, 2011). Citations, however, are used as a proxy for the assessment of impact primarily in the “publish or perish” community. To consider other disciplines and stakeholders of research, such as industry, government and academia, and in a much broader sense, the society as a whole, altmetrics (i.e. alternative, social media-based indicators) are emerging as a useful instrument to assess the “societal” impact of research data. It is assumed that altmetrics can provide a more complete picture of research uptake, besides more traditional usage and citation metrics (Bornmann 2014; Konkiel 2013). Previous work on altmetrics for research data has mainly focused on motivations for data sharing, creating reliable data metrics and effective reward systems (Costas et al. 2012).

The prerequisite to study the reuse of research data is clearly that the data has been made available to the scientific community and that it has been shared. Reuse of data can yet also mean that the creators of the data themselves, who then refer to their previous work, extensively use the data. Besides the provision and study of more technical prerequisites for data citations we argue that the processes underlying research data sharing and the attitudes towards these practice (e.g., advancing knowledge by sharing or misuse of shared data sets; Bauer et al. 2015; Fecher et al. 2015b; Tenopir et al. 2011) must also play an important role in the studies and interpretation of data citations.

Generally, Fecher et al. (2015b) found that 76 % of polled researchers believe that scientists should publish data; 88 % of respondents would actually use secondary data to perform original studies on its basis. In comparison to 2011 this is only a small increase in the results of Tenopir et al. In their survey, 83.3 % of scholars responded that they (somewhat) agree with “I would use other researchers’ datasets if their datasets were easily accessible”. In fact, the presumed visibility of research and increased reputation caused by data citations are strong drivers of data sharing practices and was stated by 79 % of respondents in the study of Fecher et al. (2015b). Tenopir et al. (2011) had 91.7 % of the researchers (somewhat) agreeing with “It is important that my data are cited when used by other researchers” and 95 % said that it is “fair to use other people’s data if there is formal citation of the data providers and/or funding agencies in all disseminated work making use of the data “(Tenopir et al. 2011, p. 10)”. Bauer et al. (2015) showed that 54 % of polled researchers consider citations to research data “as relevant scientific output in research documentation, intellectual capital report and evaluations”, although “the quality and traceability of the re-use of data” are not yet given (p. 49).

However, Fecher et al. (2015b) revealed that the majority of researchers have not shared data publicly yet (only 13 % had ever publicly shared data sets). The authors also showed that the degree of skills related to data sharing (i.e., retrieval and publication of data sets) plays a major role: “Researchers that know how to make data available to others are significantly more willing to make data available” (p. 11; see also Tenopir et al. 2011). It is not only the lack of knowledge on how to make research data available, but it is also missing information on adequate data repositories or other places to publish the data, which prevents researchers from sharing (Tenopir et al. 2011; Wallis et al. 2013).

The aforementioned studies mainly followed a qualitative approach by asking researchers about their opinions on and the frequency of data publication and sharing. Their results are therefore purely based on self-reporting. Hence, it is interesting to investigate whether the same tendencies are reflected by more quantitative evidence as provided by citation counts and altmetrics.

We consider the qualitative studies based on self-reporting as theoretical background, which will guide our interpretation of data citations and data-level metrics. The combination of both lines of research as well as the study of formal aspects of research data citations will add to the understanding of actual practices in sharing and referencing of research data, even across disciplines. It sheds light on what citation and altmetrics practices are currently in use, what types of data are actually cited and shared via social media platforms and which identifiers are popular for which data types and disciplines. Thus, the analyses will lay the foundation for the development of supporting processes and tools in research data sharing and referencing by learning, which properties make data sets successful (in terms of number of citations and altmetrics). Moreover, the study is a first approach to digging deeper into the nature and scope of Thomson Reuters Data Citation Index (DCI) as well as the research data landscape used for research assessment studies.

This study extends previous work (Peters et al. 2015) and contributes to the research on data citations in describing their characteristics as well as their impact in terms of citations and altmetrics scores. Specifically, we tackle the following research questions grouped into three thematic sets:

- Coverage and intensity of references to research data in DCI and social media channels
  - How often and to what extent are research data cited?
  - How does citedness evolve over time?
  - Are there any differences in the results of the tools used for altmetrics scores aggregation?
- Formal aspects of data citations in DCI and social media channels
  - Which identifiers are used for data citations and to what extent?
  - What are the characteristics of cited research data?
  - Which data types and disciplines are the most cited?
  - From which sources do research data originate?
- Differences in databases: DCI versus altmetrics
  - Which preferences can be observed?
  - What characteristics do uncited research data have?

## Data sources

On the Web, a large number of data repositories are available to store and disseminate research data. The Thomson Reuters Data Citation Index (DCI), launched in (2012), provides an index of high-quality research data from various data repositories across disciplines and around the world. It enables search, exploration and bibliometric analysis of research data through a single point of access, i.e. the Web of Science (Torres-Salinas et al. 2013b). The selection criteria are mainly based on the reputation and characteristics of the repositories.^4^ Three document types are available in the DCI: data set, data study, and repository. The document type “repository” can distort bibliometric analyses, because repositories are mainly considered as a source, but not as a document type.

First coverage and citation analyses of the DCI have been performed April–June 2013 by the EC3 bibliometrics group of Granada (Torres-Salinas et al. 2014; Torres-Salinas et al. 2013a). According to these studies, the data is highly skewed: Science areas accounted for almost 80 % of records in the database and four repositories contained 75 % of all the records in the database. 88 % of all records remained uncited. In Science, Engineering and Technology, citations are concentrated among datasets, whereas in the Social Sciences and Arts and Humanities, citations normally refer to data studies.

Since these first analyses, DCI has been constantly growing, now indexing nearly two million records from high-quality repositories around the world. One of the most important enhancements of the DCI has undoubtedly been the inclusion of “figshare”^5^ as new data source, which led to an increase of almost a half million of data sets and 40,000 data studies (i.e. about one-fourth of the total coverage in the database).

In contrast to the DCI, where citation information is already summarized, gathering altmetrics data is quite laborious since they are spread over a variety of social media platforms which each offer different applications programming interfaces (APIs). Tools, which collect and aggregate these altmetrics data come in handy and are now fighting for market shares since also large publishers increasingly display altmetrics for articles (e.g., Wiley^6^). There are currently three big altmetrics data providers: ImpactStory,^7^ Altmetric.com, and PlumX.^8^ Whereas Altmetrics.com and PlumX focus more on gathering and providing data for institutions (e.g., publishers, libraries, or universities), ImpactStory’s target group is the individual researcher who wants to include altmetrics information in her CV.

ImpactStory is a web-based tool which works with individually assigned permanent identifiers (such as DOIs, URLs, PubMed IDs) or links to ORCID, Figshare, Publons, Slideshare, or Github to auto-import new research outputs like e.g. papers, data sets, slides. Altmetric scores from a large range of social media platforms, including Twitter, Facebook, Mendeley, Figshare, Google + , and Wikipedia,^9^ can be downloaded as.json or.csv (as far as original data providers allow data sharing^10^). With Altmetric.com, users can search within a variety of social media platforms (e.g., Twitter, Facebook, Google+, or 8000 blogs^11^) for keywords as well as for permanent identifiers (e.g., DOIs, arXiv IDs, RePEc identifiers, handles, or PubMed IDs). Queries can be restricted to certain dates, journals, publishers, social media platforms, and Medline Subject Headings. The search results can be downloaded as.csv from the Altmetric Explorer (web-based application) or via the API. Plum Analytics or PlumX (the fee-based altmetrics dashboard) offers article-level metrics for so-called artifacts, which include articles, audios, videos, book chapters, or clinical trials.^12^ Plum Analytics works with ORCID and other user IDs (e.g., from YouTube, Slideshare) as well as with DOIs, ISBNs, PubMed-IDs, patent numbers, and URLs. Because of its collaboration with EBSCO Plum Analytics can provide statistics on the usage of articles and other artifacts (e.g., views to or downloads of html pages or pdfs), but also on, amongst others, Mendeley readers, GitHub forks, Facebook comments, and YouTube subscribers.

## Methodology

In our work, we used DCI to retrieve records of cited research data. First, we conducted a general analysis of citedness among all items published in the last five and a half decades (1960–1969, 1970–1979, 1980–1989, 1990–1999, 2000–2009, and 2010–2014) (*n* = 3,984,028 items). Then, we downloaded and analysed all items with two or more citations (Sample 1, *n* = 10,934 records). Since the study’s focus was on the actual reuse of data, we limited our analysis to research data that have been cited at least twice in order to reduce the effect of self-citations generated by single papers produced on the basis of the particular data set (the DCI does not report the number of self-citations). In contrast to the work of Torres-Salinas et al. (2013b), the present study does not aim at making general conclusions about the entire DCI, but deliberately uses the subset of more frequently cited data sets. The following metadata fields were used in the analysis: available DOI or URL, document type, source, research area, publication year, data type, number of citations and ORCID availability.^13^ Then, the citedness in the database was computed for each decade considered in this study and analysed in detail for each year since 2000. Afterwards, we analysed the distribution of document types, data types, sources and research areas with respect to the availability or non-availability of the permanent identifier DOI reported by DCI.

After this, all research data with two or more citations and with an available DOI (*n* = 2907 items) were analysed with PlumX, ImpactStory, and Altmetric.com. The coverage on social media platforms and the altmetric scores obtained from all three tools were analysed and compared. Finally, all other items with two or more citations and an available URL (*n* = 8027 items) were also analysed in PlumX, the only tool enabling analyses based on URLs, and the results were compared with the ones obtained for items with a DOI.

We also analysed the distribution of document types, data types, sources and research areas (i.e. disciplines) for all research data with two or more citations and at least one altmetric score (Sample 2; *n* = 301 items) with respect to the availability or non-availability of the permanent identifier DOI reported by DCI (items with DOI and URL or items with URL only).

Since several studies on research papers showed that citations do only moderately correlate with altmetric scores (i.a. Haustein et al. 2014a, b; Schlögl et al. 2014), we investigated the relationship between citations and altmetrics for research data as well. To this end, we examined whether uncited research data is better represented in PlumX or whether discipline specific differences in citation and altmetric counts exist. Hence, we analysed the availability of both citations and altmetrics for all research data published between 2011 and 2013 in four selected disciplines (Astronomy and Astrophysics, Chemistry, Mathematics, Sociology) to determine discipline-specific dependencies (Sample 3; *n* = 1276; 991, 125 and 1662 items respectively for each discipline, total = 4054 items) and to verify whether cited research data have more and higher altmetrics scores than uncited research data. The four disciplines were chosen because they are well comparable to the categories available in figshare, the largest research data provider in DCI. Since research showed (Haustein et al. 2014c) that recent publications are better covered on social media platforms, favour was given to the last full 3 years from time of data collection (i.e. December 2014) although this leaves us with a comparatively small citation window.

## Results and discussion

### Part 1: general results

**Fig. 1:**
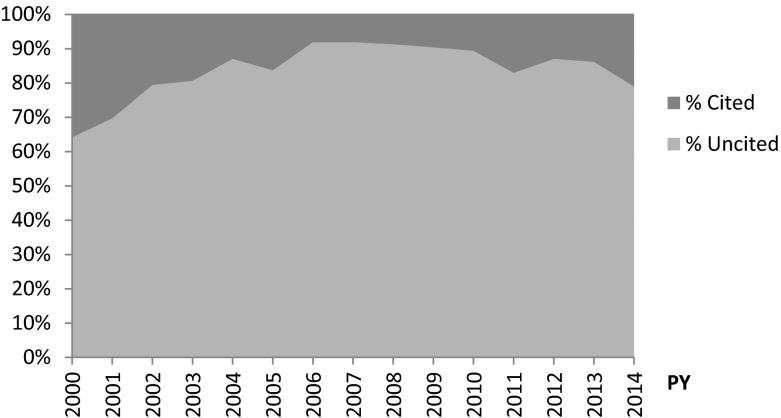
Evolution of uncitedness in DCI in the last 14 years (*n* = 3,723,691 items)

Table 1 gives an overview of the general results obtained in this study. The amount of research data available in the DCI as well as the total number of research data that has been cited at least once has increased over the last decades. Our analysis, however, revealed a high level of uncitedness of research data, which corresponds to the findings of Torres-Salinas et al. (2013b). A more detailed analysis for each year (see Fig. 1) shows, however, that the citedness is comparatively higher for research data published in recent years although the citation window is shorter. Many research data published after 2007 have been attracting citations.

**Table 1:** General description of the citation and altmetrics analyses performed in DCI for the last 5 and half decades (n = 3,984,028 items)

Data Citation Index	1960–19669	1970–19779	1980–19889	1990–19999	2000–20009	2010–2014
Total # items	6040	23,712	43,620	186,965	2,096,023	1,627,668
Uncited (%)	99.9 %	82.3 %	82.8 %	76.6 %	88.6 %	86.6 %
# Items with at least 1 citation	5	4207	7519	43,749	239,867	218,440
# Items with ≥2 citations	5	110	360	956	4727	4777
Items with ≥2 citations and DOI	4	107	343	846	1381	226
% with ≥2 citations and DOI	0.8	97.27 %	95.28 %	88.49 %	29.22 %	4.73 %
Thereoff with data in PlumX	1	5	14	40	114	20
% thereoff with data in PlumX	25.0 %	4.7 %	4.1 %	4.7 %	8.3 %	8.8 %
Items with ≥2 citations and URL only	1	3	17	110	3346	4551
% with ≥ 2 citations and URL only	0.2	2.73 %	4.72 %	11.51 %	70.78 %	95.27 %
Thereoff with data in PlumX	1	1	8	11	54	33
% thereoff with data in PlumX	100.0 %	33.3 %	47.1 %	10.0 %	1.6 %	0.7 %

The results also show a very low percentage of altmetrics scores available for research data with two or more citations (see Table 1). But in this case, two different trends can be observed: the percentage of data with DOI referred to on social media platforms is steadily increasing while the percentage of data with just a URL is steadily decreasing in the same time frame.

Interestingly, since 1990, there has been a strong rise in the number of research data, which can be referred to via URLs (mean = 320.24 items per year, min = 2 items, max = 3809 items in 2012). This URL-referenced research data also gained the most citations in total (58,285 citations, mean = 2331.4 citations per year, min = 4 citations, max = 15,868 citations for 2010). This corresponds to the results of Belter (2014) but, nevertheless, is surprising given extensive DOI promotion initiatives (e.g. the DataCite project), which resulted in a total of 2453 items (mean = 98.12 items, min = 2 items, max = 210 items in 2010) and of 47,190 citations for data sets published between 1990 and 2014 (mean = 1887.6 citations per year, min = 4, max = 7424 citations for 1999).

The percentage of research data with DOI and altmetrics scores in PlumX, the tool with the highest coverage of research data found in this study, is lower than expected (ranging between 4 and 9 %) but actually has doubled for data published in the last decades, which confirms the interest in younger research data and an increase in social media activity of the scientific community in recent years.

### Part 2: results for Sample 1

Table 2 shows an overview on the citation distribution of Sample 1 (10,934 items with at least two citations in DCI) for items with a DOI or a URL separated according to the three main DCI document types (data set, data study, and repository^14^). The results reveal that almost half of the data studies already have a DOI (48.9 %), but only few data sets do so. Data studies are on average more often cited than data sets (17.5 vs. 3.2 citations per item), and data studies with a DOI attract on average more citations than those with a URL (20 vs. 14 mean citations per item).

**Table 2:** Citation distribution of Sample 1 (*n* = 10,934 items)

Items with at least 2 citations	Document type	# Items	Total citations	Mean citations	Maximum citations	SD	Variance
All	Data set	5641	17,984	3.19	121	3.38	11.46
	Data study	5242	91,623	17.48	1236	50.22	2521.67
	Repository	51	10,076	197.57	3193	618.73	382,824.45
	Total	10,934	119,683	10.95	3193	56.39	3179.49
With DOI	Data set	342	977	2.86	52	3.86	14.93
	Data study	2565	53,293	20.78	1236	63.44	4024.45
	Total	2907	54,270	18.67	1236	59.88	3585.92
With URL only	Data set	5299	17,007	3.21	121	3.35	11.23
	Data study	2677	38,330	14.32	272	32.59	1062.31
	Repository	51	10,076	197.57	3193	618.73	382,824.45
	Total	8027	65,413	8.15	3193	54.80	3003.30

The number of repositories in the data set was low with a total number of 51. “Repository” is the document type, which attracts the most citations per item. This finding is in line with the results of Belter (2014) who also found aggregated data sets—Belter calls them “global-level data sets”—to be more cited. However, such citing behaviour has a negative side effect on repository content (i.e., the single data sets), since it is not properly attributed in favour of citing the repository as a whole.

The high values of SD and variance illustrate the skewness of the citation distribution (see Figure 1 in Peters et al. 2015). Almost half of the research data (4974 items; 45.5 %) have only two citations. Six items, two repositories and four data studies, from different decades (PY = 1981, 1984, 1995, 2002, 2011, and 1998 sorted by descending number of citations) attracted more than 1000 citations and were responsible for almost 30 % of the total number of citations.

Considering their origin, considerable differences were also reported in Sample 1 for items with or without a DOI (see Table 3). All twice or more frequently cited research data with a DOI are archived in only nine repositories, while 92 repositories are responsible for research data without a DOI.

**Table 3:** Analysis of Sample 1 by sources (repositories) (*n* = 10,934 items)

Data types (with DOI)	# Items	# Citations	Data types (with URL only)	# Items	# Citations
Inter-university Consortium for Political and Social Research	2530	53,041	miRBase	3456	10,209
Worldwide Protein Data Bank	229	458	Cancer Models Database	864	2698
Oak Ridge National Laboratory Distributed Active Archive Center for Biogeochemical Dynamics	108	508	UK Data Archive	836	25,479
Archaeology Data Service	21	75	European Nucleotide Archive	361	1346
3TU.Datacentrum	8	22	Gene Expression Omnibus	353	754
SHARE—Survey of Health, Ageing and Retirement in Europe	4	151	National Snow and Ice Data Center	298	2796
World Agroforestry Centre	3	6	Australian Data Archive	264	2469
Dryad	2	4	Australian Antarctic Data Centre	249	1621
GigaDB	2	5	nmrshiftdb2	219	445
			Finnish Social Science Data Archive	183	913

Table 4 shows the top 10 repositories with regard to the number of items. Considering the number of citations, there are three other repositories, which account for more than 1000 citations each: Manitoba Centre for Health Policy Population Health Research Data Repository (29 items with a total of 1631 citations), CHILDES—Child Language Data Exchange System (one item with 3082 citations), and World Values Survey (one item with 3193 citations). Interestingly, although “figshare” accounts for almost 25 % of the DCI, no item from “figshare” was cited at least twice in DCI. For a more in-depth analysis of figshare see Kraker et al. (2015). We also noted that the categorization of figshare items is missing. All items are assigned to the Web of Science category (WC) “Multidisciplinary Sciences” or the Research Area (SU) “Science and Technology/Other Topics” preventing detailed topic-based citation analyses. Furthermore, only nine items from Sample 1 were related to an ORCID, three data sets with a DOI, and three data sets and data studies with a URL.

**Table 4:** Analysis of Sample 1 by data types (manually merged), top 10 types (*n* = 10,934 items)

Data types (with DOI)	# Items	# Citations	Data types (with URL only)	# Items	# Citations
Survey data	1734	43,686	Sequence data	3408	10,458
Administrative records data	302	3326	Profiling by array, gen, etc	352	752
Aggregate data	274	9440	Individual (micro) level	240	9024
Event/transaction data	210	2400	Numeric data	216	4317
Clinical data	118	3469	Structured questionnaire	155	673
Census/enumeration data	109	1019	Survey data	127	1315
Protein structure	95	190	Seismic:Reflection:MCS	47	185
Observational data	30	575	Statistical data	41	1352
Program source code	10	116	Digital media	40	290
Roll call voting data	8	236	EXCEL	25	101

Table 4 also shows that there are big differences between the most cited data types when considering research data with a DOI or just a URL. Survey data, aggregate data, and clinical data are the most cited ones of the first group (with a DOI), while sequence data and numerical and individual level data are the most cited data types of the second group (with a URL). Apart from survey data, there is no overlap in the top 10 data types indexed in DCI. Similar results were obtained when considering data sets and data studies separately.

Disciplinary differences become apparent in the citations of DOIs and URLs as well as in the use of certain document types. As shown in Table 5, it is more common to refer to data studies via DOIs in the Social Sciences than in the Natural and Life Sciences, where the use of URLs for both data studies and data sets is more popular. These findings confirm the results from Torres-Salinas et al. (2014). The authors report that citations in Science, Engineering and Technology citations are concentrated on data sets, whereas the majority of citations in the Social Sciences and Arts and Humanities refer to data studies. The results of Table 5 suggest that these differences could be simply related to the availability of a DOI.

**Table 5:** Analysis of Sample 1 by research areas and document types, top 10 areas (*n* = 10,934 items)

With DOI					With URL only				
Research area	# Items		# Citations		Research area	# Items	# Citations		
	Data set	Data study	Data set	Data study		Data set	Data study	Data set	Data study
Criminology and Penology		471		4403	Genetics and Heredity	4658	159	14,024	571
Sociology		432		7930	Meteorology and Atmospheric Sciences	91	298	493	2796
Government and Law		352		10,399	Biochemistry and Molecular Biology; Genetics and Heredity		353		754
Demography		317		9178	Sociology		286		1994
Health Care Sciences and Services		290		8170	Physics	5	214	10	435
Biochemistry and Molecular Biology	229		458		Business and Economics; Sociology		143		12,665
Business and Economics		204		3083	Biochemistry and Molecular Biology; Spectroscopy	129		383	
Environmental Sciences and Ecology; Geology	108		508		Oceanography; Geology	114		353	
Education and Educational Research		69		1881	Demography; Sociology		103		5673
Family Studies		68		2268	Sociology; Demography; Communication		84		393
Sum	337	2203	966	47,312	Sum	4997	1640	15,263	25,281

### Part 3: results for Sample 2

Sample 2 comprises all items from DCI satisfying the following criteria: two or more citations in DCI, a DOI or a URL and at least one altmetrics score in PlumX (*n* = 301 items).

Table 6 shows the general results for this sample. The total number of altmetrics scores is lower than the number of citations for all document types with or without a DOI. Furthermore, the mean altmetrics score is higher for data studies than for data sets.

**Table 6:** Citation and altmetrics results of Sample 2 (*n* = 301 items) according to document type

Document type	# Items	Total citations	Mean citations	Maximum citations	SD	Variance
With DOI						
Data set	15	173	11.53	52	13.75	189.12
Data study	179	6716	37.52	1135	107.36	11,525.43
Total	194	6889	35.51	1135	103.40	10,691.82

Document type	# Items	Total scores	Mean scores	Maximum scores	SD	Variance
With DOI						
Data set	15	34	2.27	6	1.75	3.07
Data study	179	710	3.97	64	7.42	55.09
Total	194	752	376.00	748	526.09	276,768.00

Document type	# Items	Total citations	Mean citations	Maximum citations	SD	Variance
With URL only						
Data set	24	172	7.17	46	10.12	102.41
Data study	31	779	25.13	272	51.67	2669.65
Repository	44	9677	219.93	3193	662.92	439,464.20
Total*	99	10,628	107.35	3193	451.61	203,954.50

Document type	# Items	Total scores	Mean scores	Maximum scores	SD	Variance
With URL only						
Data set	24	428	17.83	378	76.75	5890.23
Data study	31	664	21.42	213	53.25	2835.65
Repository	44	3961	90.02	1150	198.53	39,415.70
Total*	99	5319	49.71	1150	139.82	19,549.38

* 8 items with URL that were found in PlumX could not properly be identified (broken URL, wrong item, etc.)

Tables 7 and 8 show the distributions of data types and subject areas in this sample. Most data with DOI are survey data, aggregate data, event over transaction data, whereas sequence data and images are most often referred to via URL only (see Table 6). Microdata with DOI and spectra with URL only are the data types with the highest altmetrics scores per item.

**Table 7:** Citation and altmetrics overview of Sample 2 (*n* = 301 items) according to their data type

Data type (with DOI)	# Items	Total citations	Mean citations	Total scores	Mean scores	Data type (with URL only) *	# Items	Total citations	Mean citations	Total scores	Mean scores
Survey data	110	5276	47.96	353	3.21	miRNA sequence data	15	71	4.73	21	1.40
Aggregate data	26	793	30.50	80	3.08	FITS images; spectra; calibrations; redshifts	4	248	62	16	4.00
Event/transaction data	19	414	21.79	43	2.26	Statistical data	3	333	111	22	7.33
Administrative records data	13	125	9.62	58	4.46	Expression profiling by array	3	6	2	4	1.33
Clinical data	11	314	28.55	26	2.36	Sensor data; survey data	2	51	25.5	10	5.00
Census/enumeration data	8	90	11.25	14	1.75	Quantitative	2	35	17.5	10	5.00
Observational data	4	99	24.75	7	1.75	Images	1	20	20	3	3.00
Longitudinal data; Panel Data; Micro data	2	79	39.50	46	23.00	Images; spectra	1	4	4	102	102.00
Roll call voting data	2	178	89.00	3	1.50	Table	1	9	9	1	1.00
Machine-readable text	1	5	5.00	1	1.00	Redshifts; spectra	1	5	5	213	213.00
Program source code	1	2	2.00	1	1.00	Images; spectra; astrometry	1	2	2	90	90.00

Field DY; no aggregated counts, without consideration of the “document type” “repository” = 34 items

**Table 8:** Citation and altmetrics overview of Sample 2 (*n* = 301 items) according to their subject area

With DOI				With URL only			
Subject areas	# Items	# Citations	# Scores	Subject areas	# Items	# Citations	# Scores
Sociology	35	1226	213	Genetics and Heredity	26	492	654
Government and Law	28	793	53	Meteorology and Atmospheric Sciences	15	166	28
Criminology and Penology	22	317	42	Astronomy and Astrophysics	9	933	427
Health Care Sciences and Services	14	1498	70	Biochemistry and Molecular Biology; Genetics and Heredity	5	22	557
Environmental Sciences and Ecology; Geology	14	171	33	Cell Biology	4	13	383
Demography	12	433	28	Health Care Sciences and Services; Business and Economics	3	335	68
Family Studies	10	166	26	Genetics and Heredity; Biochemistry and Molecular Biology	2	27	36
Archaeology	10	47	139	Business and Economics	2	35	10
Education and Educational Research	9	661	40	Health Care Sciences and Services	2	423	2
International Relations	9	384	46	Communication; Sociology; Telecommunications	2	51	10

In terms of subject areas the results of Table 8 are very similar to the results of Table 5. Taking into account the small sample size, however, it is notable that in some subject areas (e.g. Archaeology or Cell Biology) research data receive more interest on social media platforms, reflected by altmetrics scores, than via citations in traditional publications. This is also confirmed by the missing correlation between citations and altmetrics scores for this sample (see Figure 2 in Peters et al. 2015). In both cases it becomes clearly apparent that altmetrics can complement traditional impact evaluation.

Nevertheless, coverage of research data on social media platforms is still low, e.g. from the nine repositories whose data studies and data sets were cited twice in DCI and had a DOI (see Table 3), only five items had altmetrics scores in PlumX, and only one DOI item of Sample 2 included an ORCID.

### Part 4: selected altmetrics scores and comparison of the results of three altmetrics tools

Table 9 shows the general results obtained in PlumX according to the aggregation groups used in this tool (captures, social media, mentions, and usage) for all document types and with or without DOI.

**Table 9:** PlumX altmetrics scores for all document types in Sample 2 (*n* = 301 items) with or without DOI

	Document type	With DOI			With URL only			
		Data set	Data study	Total	Data set	Data study	Repository	Total
	# Items	15	179	194	24	31	44	99
Captures	Sum	32	471	503	0	0	30	30
	Mean	2.13	2.63	2.59	0.00	0.00	0.68	0.28
	Max	6	48	48	0	0	23	23
Social media	Sum	1	220	221	407	281	3060	3890
	Mean	0.07	1.23	1.14	16.96	9.06	69.55	36.36
	Max	1	58	58	366	119	1008	1008
Mentions	Sum	1	13	14	13	62	433	629
	Mean	0.07	0.07	0.07	0.54	2.00	9.84	5.88
	Max	1	4	4	12	31	119	120
Usage	Sum	0	6	6	8	321	438	770
	Mean	0.00	0.03	0.03	0.33	10.35	9.95	7.20
	Max	0	6	6	4	187	92	187
Total entries		34	710	744	428	664	3961	5319
% Captures		94.1	66.3	67.6	0.0	0.0	0.8	0.6
% Social media		2.9	31.0	29.7	95.1	42.3	77.3	73.1
% Mentions		2.9	1.8	1.9	3.0	9.3	10.9	11.8
% Usage		0.0	0.8	0.8	1.9	48.3	11.1	14.5

While DOIs for data sets seem to be important in order to get captures (mainly in Mendeley), a URL is sufficient for an inclusion in social media tools like Facebook, Twitter, etc. In Peters et al. (2015), it has been shown that cited research data with DOI attracting two or more citations and with at least one entry in PlumX gain more citations than altmetrics scores, and that there is no correlation between highly cited and highly altmetrics-scored research data. Altmetrics scores as reported by PlumX for the top 10 research data-URLs with two or more citations are depicted in Table 10. Research data-URLs receive far more citations in total and also significantly higher altmetrics scores than research data with DOIs, especially when we compare mentions and social media.

**Table 10:** Top 10 research data with URL only according to the total scores as reported in PlumX

Title	PY	Data type	Total captures	Total mentions	Total social media	Total usage	Total scores	Total citations
DrugBank	2006	Repository	0	119	1008	23	1,150	3
http://www.uniprot.org	2002	Repository	0	91	379	68	538	11
WVS Database	1981	Repository	0	19	358	7	384	3193
The Cell: An Image Library—Image CIL:12654	2012	Data set	0	12	366	0	378	2
Home | 1000 Genomes	2008	Repository	0	32	222	92	346	344
CDC—BRFSS—Behavioral Risk Factor Surveillance System	1984	Repository	0	21	160	68	249	13
BOSS: Dark Energy and the Geometry of Space—SDSS-III	2011	Data study	0	31	119	63	213	5
http://bit.ly/kHkfW			0	120	81	0	201	
Genotype information for Agrostis chloroplast SSR, matK, and Agrostis nuclear SSR markers	2012	Data study	0	0	0	0	187	2
Human Metabolome Database	2005	Repository	0	17	134	16	167	3

The comparison of altmetrics aggregation tools also revealed that ImpactStory only found Mendeley reader statistics for research data: 78 DOIs had 257 readers. Additionally, ImpactStory found one other DOI in Wikipedia. ImpactStory found five items, which have not been found by PlumX, although they both relied on the same data source (Mendeley); the Mendeley data scores were exactly the same in PlumX and in ImpactStory.

PlumX found 18 items that were not available via ImpactStory. These research data were distributed on social media platforms (mostly shares in Facebook) and one entry has been used via click on a Bitly-URL. The tool Altmetric.com found only one of 194 items.

As already reported in previous analyses (Jobmann et al. 2014), PlumX is the tool with the highest coverage of research products found on social media platforms. Whereas Mendeley is well covered in ImpactStory, no other social media metrics were found for the data set used in this study.

### Part 5: results for Sample 3

Table 11 presents the amount of and the citation numbers for different types of research data published between 2011 and 2013. Most of the research data is reported for Sociology, which is also the discipline with the most citations in total. Similar to the results reported above, the percentage of research data with DOI varies considerably between the disciplines covered by the DCI: in Astronomy and Astrophysics, <1 % of research data come with a DOI, whereas in Sociology and Mathematics the percentage ranges between 10 % and 14 %. DOIs are most prominent in Chemistry where 38 % of research data have a DOI. Also, disciplinary differences in the assignment of DOIs to research data types become apparent. In Astronomy and Astrophysics and in Chemistry only data studies have a DOI, while in Mathematics and Sociology, there are more data sets than data studies with a DOI.

**Table 11:** Citation numbers for research data published between 2011 and 2013 in four selected disciplines (Sample 3; *n* = 4054 items)

Subject category	Citation analysis PY = 2011–2013						
	DT	All	# Items	# Citations	Citations/item	Max	SD
Astronomy and Astrophysics	All DTs	Data set	1162	2	0.00	1	0.041
		Data study	106	84	0.79	5	0.765
		Repository	8	0	0.00	0	0.000
		Total	1276	86	0.07	5	0.312
	With DOI	Data study	4	1	0.25	1	0.500
		Total	4	1	0.25	1	0.500
	Without DOI	Data set	1162	2	0.00	1	0.041
		Data study	102	83	0.81	5	0.767
		Repository	8	0	0.00	0	0.000
		Total	1272	85	0.07	5	0.311
Chemistry	All DTs	Data study	990	22	0.02	1	0.147
		Repository	1	0	0.00	0	
		Total	991	22	0.02	1	0.147
	With DOI	Data study	373	22	0.06	1	0.236
		Total	373	22	0.06	1	0.236
	Without DOI	Data study	617	0	0.00	0	0.000
		Repository	1	0	0.00	0	
		Total	618	0	0.00	0	0.000
Mathematics	All DTs	Data set	120	0	0.00	0	0.000
		Data study	5	1	0.20	1	0.447
		Total	125	1	0.01	1	0.089
	With DOI	Data set	12	–	0.00	0	0.000
		Data study	5	1	0.20	1	0.447
		Total	17	1	0.06	1	0.243
	Without DOI	Data set	108	0	0	0	
		Total	108	0	0	0	
Sociology	All DTs	Data set	881	12	0.01	4	0.165
		Data study	781	181	0.23	41	1.645
		Total	1662	193	0.12	41	1.139
	With DOI	Data set	117	0	0	0	
		Data study	56	46	0.82	5	1.177
		Total	173	46	0.27	5	0.769
	Without DOI	Data set	764	12	0.02	4	0.177
		Data study	725	135	0.19	41	1.668
		Total	1489	147	0.10	41	1.173

The investigation of the citation activity reveals that the total number of citations, as well as the mean and maximum values, are very low across all disciplines. In Astronomy and Astrophysics, 94 % of the research data remain uncited, in Chemistry it is 98 %, in Mathematics 99 % and in Sociology 95 %. This is in line with the results of the analyses of the other samples in this study and may be due to the short citation window. Discipline-specific citation behaviour is also visible: in Mathematics and Chemistry only research data with DOIs are cited, whereas in the other disciplines both DOIs and URLs are used for citation of research data. Sociology shows the highest citation activity, where a particular data study has attracted more than 40 citations, despite the short citation window.

In terms of altmetrics scores, research data from Astronomy and Astrophysics have the greatest impact (see Table 12) and data sets receive far higher altmetrics scores than data studies—although we should consider that interpretation of altmetrics scores is only based on a very low number of research data available on social media platforms (see column “items with scores” in Table 12). Interestingly, research data without DOIs gain the highest altmetrics scores in all disciplines. Research data without DOIs from Sociology only receive mentions, which are exclusively derived from Facebook comments.

**Table 12:** Altmetrics scores for research data published between 2011 and 2013 in four selected disciplines (Sample 3)

Subject category	Altmetric analysis in PLUM-X								
	Items with scores	Data type	With DOI	Total captures	Total mentions	Total usage	Total social media	Total scores	Total citations
Astronomy and Astrophysics	1	Data set*	No	0	114	32	477	623	0
	2	Data set	No	0	31	125	106	262	0
	3	Repository*	No	0	31	63	119	213	0
	4	Data set*	No	0	10	54	38	102	4
	5	Data set*	No	0	7	7	75	89	0
	6	Data study	No	0	0	7	0	7	0
	7	Data study	Yes	0	0	0	3	3	1
	8	Data study	No	0	0	0	2	2	0
	9	Data study	No	0	0	0	2	2	0
	10	Data study	No	0	0	0	1	1	0
	11	Data study	Yes	0	0	0	1	1	0
	12	Data set	No	0	0	0	1	1	0
Chemistry	0	n.a.	n.a.	0	0	0	0	0	0
Mathematics	1	Data set	No	0	0	0	2	2	0
Sociology	1	Data set*	No	0	11	0	0	11	0
	2	Data study	No	0	4	0	0	4	0
	3	Data set*	No	0	4	0	0	4	0
	4	Data set*	No	0	4	0	0	4	0
	5	Data set*	No	0	2	0	0	2	0
	6	Data set*	No	0	2	0	0	2	0
	7	Data set*	No	0	2	0	0	2	0
	8	Data study*	No	0	2	0	0	2	0
	9	Data study*	No	0	1	0	0	1	0
	10	Data set*	No	0	1	0	0	1	0

* Matching of source information from DCI (i.e. URL and title of research data) and result from PlumX is not necessarily correct because of missing or changed information in altmetrics search results. Since URLs are not permanent identifiers like DOIs URLs as indexed in the DCI may have disappeared or changed and, thus, PlumX might not have retrieved the exact same content as has been indexed by the DCI

When comparing the results of Tables 11 and 12, the same tendencies are revealed as in research paper citations and altmetrics scores (e.g., Haustein et al. 2014a). For example, eight repositories in Astronomy and Astrophysics have not been cited at all, but three of them received together an altmetrics score of 213. On the other hand, data sets, data studies, and repositories from Sociology receive 193 citations in sum, but an altmetrics score of only 33. Mathematical research data are neither cited nor present on social media platforms.

## General conclusions

### Coverage and intensity of references to research data in DCI and social media channels

Most of the research data still remain uncited (approx. 86 %) and total altmetrics scores found via aggregation tools are even lower than the number of citations. However, research data published from 2007 onwards have gradually attracted more citations reflecting a bias towards more recent research data which might be due to the awareness of and demand for research data reuse (Fecher et al. 2015a).

The disciplinary analysis showed that altmetrics scores vary between disciplines at a low level; a very limited amount of research data (<1 % in each discipline) received any altmetrics scores in these disciplines at all. Only a number of research data from Astronomy and Astrophysics has received scores across various sources.

### Formal aspects of data citations in DCI and social media channels

In the DCI, the availability of cited research data with a DOI is rather low. A reason for this may be the increase of available and indexed research data in recent years. Furthermore, the percentage of cited research data with a DOI has not increased as expected, which indicates that citations do not depend on this standard identifier in order to be processed by the DCI. Nevertheless, data studies with a DOI attract more citations than those with a URL. In a nutshell, the analyses showed that there is a low number of research data with a DOI in general and that there are considerable differences in the adoption of DOIs across disciplines as well as across research data types (e.g., data studies). Surprisingly, the DOI in cited research data has so far been more embraced in the Social Sciences than in the Natural Sciences.

Furthermore our study shows an extremely low number of research data with two or more citations (only nine out of around 10,000) related to an ORCID. Only three of them had a DOI likewise. This illustrates that we are still a far cry from the establishment of permanent identifiers and their optimal interconnectedness in a data source.

The qualitative studies on data sharing (Tenopir et al. 2011; Wallis et al. 2013) already showed that there are many uncertainties regarding sharing and reuse of research data on the researchers’ side. Hence, we may argue that differences in URL and DOI citation behaviour as well as the lack of data citations at all might be due to the lack of knowledge on how to formally refer to data sets as well as on how to find reusable data sets. The lack of standardized data citation practices is even more problematic considering that there is often more than one research product associated with a data set. There are, however, first approaches towards citation standards, e.g. the American Psychological Association (APA) recommends to use the DOI first and then the URL—depending on the availability.^15^ They argue that the DOI is preferable to the URL since the DOI is a persistent identifier.^16^ A more speculative explanation—which needs to be backed up with future research, but is informed by the disciplinary differences in URL-/DOI-use—is that the different practices of citing URLs or DOIs may point to the researchers’ different concepts of the referenced scientific products, in this case research data. This investigation might be especially useful by applying disciplinary lenses.

### Differences in databases: DCI versus altmetrics

No correlation between citation and altmetrics scores could be observed in our preliminary analysis: neither the most cited research data nor the most cited sources (repositories) received the highest scores in the altmetrics aggregator PlumX. The low percentage of altmetrics scores for research data with two or more citations corroborates a threefold hypothesis: First, research data are either rarely published or not findable on social media platforms, because DOIs or URLs are not used in references thus resulting in a low coverage of items. Second, research data are not widely shared on social media by the scientific community so far which would result in higher altmetrics scores.^17^ Third, the reliability of altmetrics aggregation tools is questionable as the results on the coverage of research data on social media platforms differ widely between tools. However, the steadily increasing percentage of cited research data with a DOI suggests that the adoption of this permanent identifier increases the online visibility of research data and may intensify inclusion in altmetrics tools (since they heavily rely on DOIs or other permanent identifiers for search) and in (automated) referencing practices on social media platforms.

The first finding is in line with other studies on correlations between altmetrics and citations to research papers (amongst others: Thelwall et al. 2013) resulting only in low or moderate values. It is possible, however, that this finding is an artefact of our initial data collection limiting the studied data set to research data with at least two citations. The analysis of cited and uncited research data in four different disciplines showed that certain research data can get high altmetrics scores when having no citations and no DOIs. It seems that at the moment two different types of referencing practices on social media platforms exist. Presumably, DOI referencing practices and social media-activities differ between communities (e.g., scientists who refer to research data via DOIs in their papers and laymen who refer to research data via URLs in their social media-posts). It is also possible that the altmetrics scores depend on the audiences of social media platforms.

### Limitations

In our opinion, our work has two limitations. Firstly, the results rely on the indexing quality of the DCI. Our analysis showed that the categorisation in DCI is problematic at times. This is illustrated by the fact that all items from figshare, which is one of the top providers of records, are categorised in “Miscellaneous”. The category “repository” is rather a source than a document type. Such incorrect assignments of data types and disciplines can easily lead to wrong interpretations in citation analyses. Furthermore, it should be taken into account that citation counts are not always traceable.

Secondly, we only take into consideration data sets cited at least two times. Given that we cut the ‘long tail’ of uncited and barely cited research data, we excluded basic statistical computation such as means, SD, correlations and regressions from our study. Hence, the conclusions we have drawn necessarily refer to frequently cited data sets, which is in line with our overarching research question on the reuse of research data: What are the quantities, formal characteristics, and origins of successfully reused data sets (i.e. cited more than once) and which differences appear in formal citations and altmetrics? Accordingly, our study is a first step towards the understanding of research data reuse and citation practices and complements the work of Torres-Salinas et al. (2013b), which has shed light on the reliability of the DCI.

Still, citations of research data should be studied in more detail. They certainly differ from citations of papers relying on these data with regard to dimension and purpose. For example, we found that entire repositories are proportionally more often cited than single data sets, which was confirmed by a former study (Belter 2014). Therefore, it will be important to study single repositories (such as figshare) in more detail. It is crucial to further explore the real meaning and rationale of research data citations and how they depend on the nature and structure of the underlying research data, e.g., in terms of data curation and awarding of DOIs.

## Footnotes

1 http://escholarship.org/uc/item/9kf081vf.

2 https://www.datacite.org/news/cern-launches-data-sharing-portal.html.

3 http://blog.impactstory.org/data-impact-metrics/.

4 http://thomsonreuters.com/data-citation-index, http://thomsonreuters.com/products/ip-science/04_037/dci-selection-essay.pdf.

5 http://figshare.com.

6 http://eu.wiley.com/WileyCDA/PressRelease/pressReleaseId-108763.html?campaign=wlytk-41414.4780439815.

7 https://impactstory.org.

8 https://plu.mx.

9 http://feedback.impactstory.org/knowledgebase/articles/367139-what-data-do-you-include-on-profiles.

10 For example, Scopus’ citation data can be displayed on the ImpactStory-profile but not downloaded.

11 http://support.altmetric.com/knowledgebase/articles/83335-which-data-sources-does-altmetric-track.

12 http://www.plumanalytics.com/metrics.html.

13 The DCI field “data type” was manually merged to more general categories; e.g. “survey data in social sciences” was merged with the category “survey data”.

14 Even if we consider repositories not really to be a document type, they were included in Table 2 to give a complete picture of the citation volume in DCI.

15 http://blog.apastyle.org/apastyle/2013/12/how-to-cite-a-data-set-in-apa-style.html.

16 http://blog.apastyle.org/apastyle/2014/07/how-to-use-the-new-doi-format-in-apa-style.html.

17 figshare recently announced a partnership with Altmetric.com which might increase the visibility of altmetrics with respect to data sharing: http://figshare.com/blog/The_figshare_top_10_of_2014_according_to_altmetric/142.
